# m^6^A Modification in Mammalian Nervous System Development, Functions, Disorders, and Injuries

**DOI:** 10.3389/fcell.2021.679662

**Published:** 2021-05-25

**Authors:** Jun Yu, Yuanchu She, Sheng-Jian Ji

**Affiliations:** ^1^Shenzhen Key Laboratory of Gene Regulation and Systems Biology, Brain Research Center, Department of Biology, School of Life Sciences, Southern University of Science and Technology, Shenzhen, China; ^2^SUSTech-HKU Joint Ph.D. Program, School of Biomedical Sciences, Li Ka Shing Faculty of Medicine, The University of Hong Kong, Hong Kong, China

**Keywords:** m^6^A modification, nervous system, development, neurological disorders, learning and memory

## Abstract

*N*^6^-methyladenosine (m^6^A) modification, as the most prevalent internal modification on mRNA, has been implicated in many biological processes through regulating mRNA metabolism. Given that m^6^A modification is highly enriched in the mammalian brain, this dynamic modification provides a crucial new layer of epitranscriptomic regulation of the nervous system. Here, in this review, we summarize the recent progress on studies of m^6^A modification in the mammalian nervous system ranging from neuronal development to basic and advanced brain functions. We also highlight the detailed underlying mechanisms in each process mediated by m^6^A writers, erasers, and readers. Besides, the involvement of dysregulated m^6^A modification in neurological disorders and injuries is discussed as well.

## Introduction

Messenger RNAs (mRNAs) play crucial roles in biological processes, which not only serve as messengers that pass genetic information from DNA to protein but also bear various post-transcriptional regulation mechanisms. Modifications on mRNA have been studied for several decades (Boccaletto et al., 2018). Other than 5′ cap and 3′ polyadenylation, numerous modified nucleotides such as *N*^6^-methyladenosine (m^6^A), *N*^1^-methyladenosine (m^1^A), *N*^6^,2′-O-dimethyladenosine (m^6^A_m_), 5-methylcytosine (m^5^C), and 5-hydroxymethylcytosine (hm^5^C) have been identified (Roundtree et al., 2017a). Modifications on mRNAs can change the structural properties of modified mRNAs, which affects the accessibility and affinity to specific RNA binding proteins (RBPs). Similar to chemical modifications on DNA and histone proteins, mRNA modifications have profound significance to biological processes.

m^6^A modification, as the most prevalent internal chemical modification on mRNA, was found more than four decades ago (Desrosiers et al., 1974; Adams and Cory, 1975; Furuichi et al., 1975; Wei et al., 1975). However, due to the lack of detection methods, functional studies on m^6^A were greatly hindered. The discovery of the first m^6^A demethylase in 2011 led to a resurgence in exploring m^6^A modification (Cao et al., 2016). Moreover, with the advances in biochemistry and sequencing technology in recent years, much progress has been achieved on m^6^A modification.

The abundance of m^6^A was estimated in a ratio of 0.1–0.4% of adenosine in mammals (about 3∼5 m^6^A modification per mRNA) (Rottman et al., 1974; Wei et al., 1975; Fu et al., 2014). It occurs on the consensus motif DRACH (D means a non-cytosine base, R refers to G/A, A is the m^6^A modified site, and H represents a non-guanine base) (Fu et al., 2014; Livneh et al., 2020). m^6^A modification is preferentially distributed in long coding exons, 3′ untranslated regions (UTR), and near the stop codon of mRNAs (Dominissini et al., 2012; Meyer et al., 2012). m^6^A has been found to be dynamically regulated and involved in many biological processes by affecting the fate of modified mRNA. In this review, we will summarize the recent findings of m^6^A modification in the nervous system from development to higher functions and from neurological disorders to injuries.

## m^6^A Writers, Erasers, and Readers

### m^6^A Writers

The deposition of m^6^A modification on mRNA is mediated by a multi-component methyltransferase complex. The methyltransferases are also called m^6^A writers, including methyltransferase-like 3 (METTL3), methyltransferase-like 14 (METTL14), and Wilms tumor 1-associated protein (WTAP) (Bokar et al., 1997; Liu et al., 2014; Ping et al., 2014; Schwartz et al., 2014). During the methylation process, METTL3 and METTL14 form a stable heterodimer complex and work synergistically to regulate adenosine methylation. METTL3 is the catalytically active enzymatic component, while METTL14 is an allosteric activator (Sledz and Jinek, 2016; Wang P. et al., 2016; Wang X. et al., 2016). This METTL3-METTL14 complex catalyzes the vast majority of m^6^A modification on mRNA, as ablation of METTL3 or inactivation of METTL14 in mouse embryonic stem cells leads to the loss of more than 99% of total m^6^A in mRNA (Geula et al., 2015). The remaining modified m^6^A residues in mRNA could be catalyzed by METTL16 or other potential methyltransferases (Zaccara et al., 2019). WTAP is a critical adaptor that translocates the METTL3-METTL14 complex into nuclear speckles, thus facilitating the methylation efficiency (Ping et al., 2014; Schwartz et al., 2014).

### m^6^A Erasers

The discovery of m^6^A erasers (demethylases) proves that m^6^A is a dynamic and reversible modification. The first m^6^A eraser, fat mass and obesity-associated (FTO), was discovered in 2011 (Jia et al., 2011). FTO belongs to the Fe (II) and α-ketoglutarate-dependent AlkB family (Gerken et al., 2007), which was initially found to be associated with body weight and food intake in mice (Fischer et al., 2009; Church et al., 2010). It can effectively demethylate m^6^A in both RNA and DNA *in vitro* (Jia et al., 2011). *In vivo*, FTO also demethylates specific mRNAs that affect neuronal signaling in the mouse brain (Hess et al., 2013). However, FTO was further found to preferentially demethylate m^6^A_m_ in the 5′ cap of mRNA (Mauer et al., 2017). Thus, more studies from the third parties would be required to solve this scientific dispute.

The second eraser of m^6^A, alkB homolog 5 (ALKBH5), was related to fertility in mice (Zheng et al., 2013). It also belongs to the Fe (II) and α-ketoglutarate-dependent AlkB family. ALKBH5 can catalyze the demethylation of m^6^A modification on mRNA both *in vitro* and *in vivo*, which influences the nuclear RNA export and metabolism (Zheng et al., 2013). Unlike FTO, ALKBH5 cannot demethylate m^6^A_m_ (Mauer et al., 2017).

### m^6^A Readers

*N*^6^-methyladenosine modification exerts its function by recruiting m^6^A-binding proteins, which are also called m^6^A readers. There are two ways of reader proteins to bind to m^6^A modification: direct binding and indirect binding. Direct binding relies on a specialized domain within the readers, which can directly recognize and bind to m^6^A. The first direct reader proteins identified were the YTH (YT521-B homology) domain-containing proteins (Dominissini et al., 2012). The YTH domain is a highly conserved RNA binding domain identified in a wide range of eukaryotes (Stoilov et al., 2002). There are three classes of YTH domain-containing proteins in mammals, including the YTH domain-containing family protein (YTHDF) family, YTH domain-containing protein 1 (YTHDC1), and YTH domain-containing protein 2 (YTHDC2) (Patil et al., 2018). The indirect reader proteins include HNRNPC, HNRNPG, HNRNPA2B1, and IGF2bp proteins, which can bind m^6^A through the mechanism of m^6^A-dependent mRNA structural change (Zaccara et al., 2019).

Transcriptome-wide binding sequencing studies of endogenous (Patil et al., 2016) or overexpressed YTH proteins (Wang et al., 2014, 2015) using crosslinking and immunoprecipitation (CLIP) experiments showed that most YTH proteins bind to the m^6^A consensus motif in mRNA. The distribution of the YTHDF family proteins’ binding sites is similar to the distribution pattern of m^6^A on mRNA (Patil et al., 2016). YTHDF proteins and YTHDC1 can recognize and selectively bind m^6^A through an aromatic cage (hydrophobic pocket) formed by three tryptophans in the YTH domain (Li et al., 2014; Luo and Tong, 2014; Theler et al., 2014; Xu et al., 2014).

#### YTHDF Family Proteins

YTHDF family proteins contain three members: YTHDF1, YTHDF2, and YTHDF3. YTHDF proteins have the same binding specificity toward m^6^A-modified mRNA (Xu et al., 2015). These three proteins share high similarity in amino acid sequence over their entire length and are expressed mainly in the cytoplasm (Patil et al., 2018). YTHDF proteins have almost identical YTH domains at C-terminal. Apart from the YTH domain, YTHDF family proteins contain a low-complexity region with no recognizable modular protein domain and include several P/Q/N-rich domains (Patil et al., 2018). The function of this low-complexity region is to lead mRNA-YTHDF complexes to undergo liquid-liquid phase separation to different endogenous compartments, like processing bodies (P-bodies), neuronal RNA granules, or stress granules (Ries et al., 2019).

##### YTHDF1

The role of YTHDF1 was found to promote the translation efficiency of m^6^A-modified mRNA (Wang et al., 2015). It was shown that YTHDF1 plays a dual role in this process by delivering m^6^A-modified mRNA to translation machinery and enhancing translation initiation (Wang et al., 2015). This could be possibly caused by the loop structure mediated by eIF4G and the interaction between YTHDF1 and eIF3 (Wang et al., 2015).

##### YTHDF2

YTH domain-containing protein 2 was found to be implicated in enhancing the degradation of m^6^A-modified mRNA (Wang et al., 2014). In this process, YTHDF2 binds to m^6^A-modified mRNA and translocates those mRNA from the translatable pool into P-bodies, which are mRNA decay sites (Wang et al., 2014). However, other studies did not find the existence of YTHDF2 in P-bodies (Hubstenberger et al., 2017). The possible explanation is that the association between YTHDF2 and P-bodies is transient, which results in the difficulty to detect YTHDF2 in P-bodies. Another study found that YTHDF2 regulates mRNA stability by mediating mRNA deadenylation first and then translocating to P-bodies (Du et al., 2016). The N-terminal region of YTHDF2 is capable of recruiting the CCR4-NOT deadenylase complex, causing the deadenylation of mRNA (Du et al., 2016), which finally degrades mRNA. YTHDF2 also regulate endoribonucleolytic cleavage of m^6^A-modified mRNA through interaction with RNase P/MRP, which is bridged by HRSP12 (Park et al., 2019).

##### YTHDF3

The role of YTHDF3 was characterized as working together with YTHDF1 and YTHDF2 to regulate the metabolism of m^6^A-modified mRNA (Shi et al., 2017). It has a combined effect of YTHDF1 and YTHDF2, promoting both translation and decay of m^6^A-modified mRNA (Shi et al., 2017). Knockdown of YTHDF3 reduces the RNA-binding specificity of both YTHDF1 and YTHDF2 (Shi et al., 2017). Compared with YTHDF1 and YTHDF2, YTHDF3 exerts its function on the early life cycle of RNA in the cytosol (Shi et al., 2017). When m^6^A-modified mRNA is transported to the cytoplasm, it might be initially recognized by YTHDF3. The binding of YTHDF3 could then facilitate YTHDF1 binding to the mRNA and together promote translation. Subsequently, the mRNA might be bound and partitioned among YTHDF proteins and eventually recognized by YTHDF2 for degradation.

However, a recent study has argued that all the YTHDF proteins function redundantly to mediate mRNA degradation (Zaccara and Jaffrey, 2020). Thus, more studies are needed to explore their functions in detail.

#### YTHDC1

YTH domain-containing protein 1 is predominantly expressed in nuclear speckles (Hartmann et al., 1999). It has been shown that YTHDC1 mediates splicing (Xiao et al., 2016), and nuclear export of mRNA (Roundtree et al., 2017b). As active transcription occurs in nuclear speckles, YTHDC1 may bind m^6^A-modified mRNAs and affect their splicing. By recruiting pre-mRNA splicing factor SRSF3 and inhibiting SRSF10, YTHDC1 facilitates exon inclusion in target m^6^A-modified mRNA (Xiao et al., 2016). YTHDC1 can also selectively promote the transport of m^6^A-modified mRNA from nuclear to cytoplasm through interacting with nuclear mRNA receptors NXF1 and SRSF3 (Roundtree et al., 2017b). Besides, transcriptome-wide UV crosslinking immunoprecipitation (CLIP) study showed that YTHDC1 preferentially binds m^6^A residues in long non-coding RNAs (Patil et al., 2016), whereas the YTHDF family readers prefer to bind m^6^A sites on mRNA. The proper function of the long non-coding RNA, *X-inactive specific transcript (XIST*), which regulates X chromosome inactivation and transcriptional silencing of genes on the X chromosome, needs YTHDC1 to bind its m^6^A sites (Patil et al., 2016). In addition, YTHDC1 can interact with the H3K9me2 demethylase KDM3B to promote H3K9me2 demethylation and gene expression (Li et al., 2020b).

#### YTHDC2

YTH domain-containing protein 2 is expressed both in the nucleus and cytoplasm. Unlike the other m^6^A readers that are universally expressed, YTHDC2 is enriched in testes (Bailey et al., 2017; Hsu et al., 2017; Wojtas et al., 2017; Jain et al., 2018). YTHDC2 can promote the translation efficiency of its target mRNAs and also decrease mRNA levels (Hsu et al., 2017; Kretschmer et al., 2018). YTHDC2 promotes germ cell fate transition from mitosis to meiosis (Bailey et al., 2017). *Ythdc2* knockout mice show defects in spermatogenesis (Bailey et al., 2017; Hsu et al., 2017).

## The Distribution of m^6^A in the Nervous System

**FIGURE 1:**
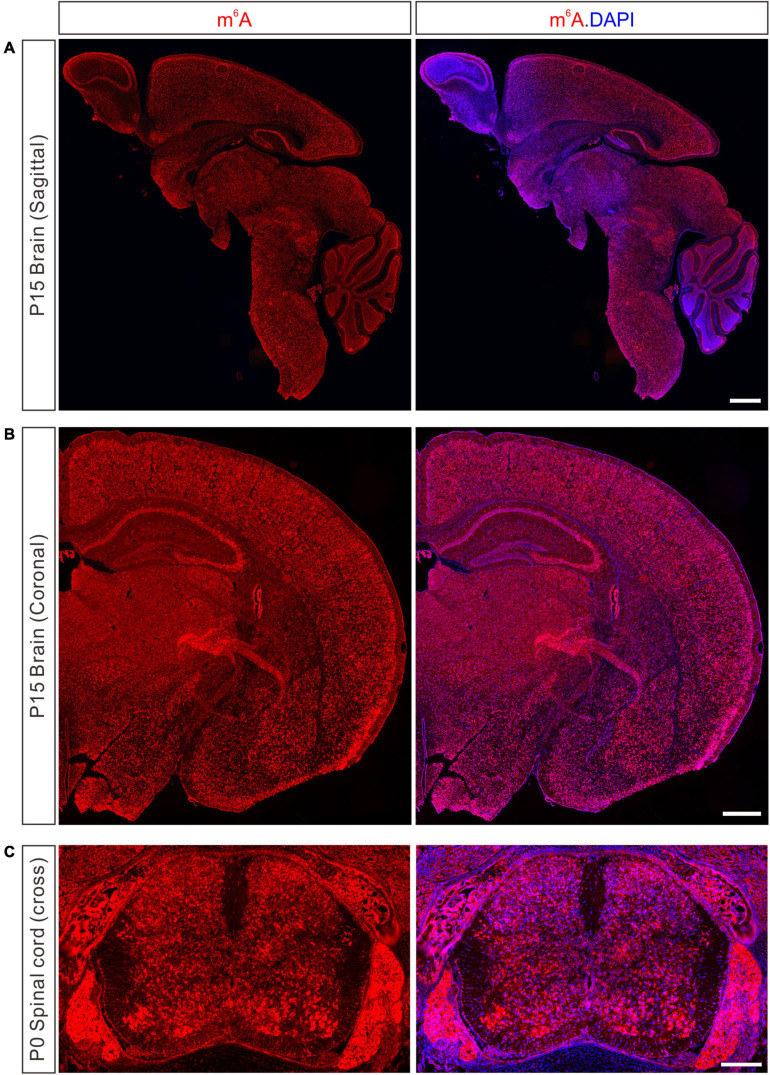
Detection of m^6^A modification in the developing nervous system of mouse. **(A)** Immunostaining of m^6^A in sagittal section of P15 mouse brain. Scale bar, 1 mm. **(B)** Immunostaining of m^6^A in coronal section of P15 mouse brain. Scale bar, 500 μm. **(C)** Immunostaining of m^6^A in cross-section of P0 mouse spinal cord and DRG. Scale bar, 200 μm.

*N*^6^-methyladenosine is widely distributed in many mouse tissues, with the highest expression in the brain (Meyer et al., 2012; Chang et al., 2017). Immunostaining with a specific m^6^A antibody revealed wide-spread and strong m^6^A signals in the developing mouse brain, spinal cord, and dorsal root ganglion (DRG) (Figure 1). Transcriptome-wide m^6^A sequencing showed that distinct m^6^A methylation patterns occur in different brain regions or at different stages in the same region, suggesting the critical dynamic involvement of m^6^A modification in neuronal development (Chang et al., 2017). In the mouse cerebral cortex and cerebellum, neurons have relatively higher m^6^A levels than glia cells (Chang et al., 2017). The highly methylated mRNAs are associated with processes such as synapse assembly and axon guidance, suggesting that m^6^A modification plays an essential role in neuronal development and brain functions (Chang et al., 2017).

## The Functions of m^6^A in the Mammalian Nervous System

For the past 5 years, tremendous progress has been made, showing that m^6^A modification can regulate multiple neuronal developmental processes and functions in mammals. We summarize these findings in Table 1 and discuss the details in the following parts.

**TABLE 1:** Roles of m^6^A modification in neuronal development and functions.

**Developmental processes and functions**	**m^6^A writers, erasers, or readers**	**Mouse models (KO, cKO) or *in vitro* (KD)**	**If cKO, which cre line?**	**Phenotype**	**Key target mRNAs identified**	**References**
Differentiation, and neurogenesis	METTL14	cKO	*Nestin-cre*	Prolonged cell cycle of radial glia cells; cortical neurogenesis extended into postnatal stages	*Pax6, Sox2, Emx2, Tbr2, Cdk9, Ccnh/Cyclin H*, and *Cdkn1C/p57*	Yoon et al., 2017
	METTL14	cKO	*Nestin-cre*	Reduced proliferation and premature differentiation of NSCs in cortex	*CBP* and *P300*	Wang Y. et al., 2018
	YTHDF2	KO	NA	Decreased proliferation and differentiation of NSPCs in cortex	*Ddr2, Mob3b, Rnf135, Speg, Flrt2, Hlf, Nrp2, Nrxn3, Ptprd*, and *Soat1*	Li et al., 2018
	FMRP	KO	NA	Delayed cell cycle and extended pool of proliferating progenitors to postnatal stages in cortex	*Ptch1, Dll1, Dlg5, Fat4, Gpr161*, and *Spop*	Edens et al., 2019
	METTL3	cKO	*Nestin-cre*	Increased apoptosis of newly generated cerebellar granule cells	*Atoh1, Cxcr4, Gli3, Jag1, Notch2, Sox2, Yap1, Dapk1, Fadd, Ngfr, Grin1, Atp2b3, Grm1*, and *Lrp8*	Wang C.X. et al., 2018
	ALKBH5	KO	NA	Aberrant proliferation and differentiation in cerebellum under hypobaric hypoxia conditions	*Letm1, Opa1*, and *Mphosph9*	Ma et al., 2018
Axon growth	FTO	KD	NA	Knockdown of FTO repressed local mRNA translation and axon growth	*GAP-43*	Yu et al., 2018
Axon guidance	YTHDF1	cKO	*Atoh1-cre*	Misprojection of pre-crossing commissural axons into motor columns of spinal cord	*Robo3.1*	Zhuang et al., 2019
Axon regeneration	METTL14	cKO	*Syn1-cre*	Reduced functional axon regeneration	*Atf3*	Weng et al., 2018
	YTHDF1	KO	NA		NR	
Synapse	YTHDF1, YTHDF3	KD	NA	KD of YTHDF1 or YTHDF3 caused abnormal dendrite spine morphology, reduced PSD95 and GluA1 expression, compromised synaptic transmission of cultured hippocampal neuron	*Apc*	Merkurjev et al., 2018
Adult neurogenesis	FTO	KO	NA	Reduced proliferation and neuronal differentiation of adult neural stem cells (aNSCs); impaired learning and memory	*Bdnf, Akt1, Akt2, Akt3*, and *S6k1*	Li et al., 2017
	FTO	cKO	*Nestin-cre*	Inhibited adult neurogenesis and neuronal development	*Pdgfra and Socs5*	Cao et al., 2020
	METTL3	KD	NA	Inhibited proliferation of aNSCs; skewed differentiation of aNSCs toward glial lineage	*Ezh2*	Chen J. et al., 2019
Gliogenesis	METTL14	cKO	*Olig2-Cre; CNP-Cre*	Loss of mature oligodendrocytes and hypomyelination	*Ptprz1* and *NF155*	Xu H. et al., 2020
	PRRC2A	cKO	*Nestin-cre; Gfap-Cre; Olig2-Cre*	Hypomyelination; locomotive and cognitive defects	*Olig2*	Wu et al., 2019
	METTL14	cKO	*Nestin-cre*	Reduced number of astrocytes in the cortex	NR	Yoon et al., 2017
	METTL3	cKO	*Nestin-cre*	Abolished scaffold organization pattern provided by Bergmann glia in cerebellum	NR	Wang C.X. et al., 2018
	YTHDF2	KO	NA	Dramatic reduction of GFAP^+^ astrocytes	NR	Li et al., 2018
Learning and memory	FTO	KD	NA	KD of FTO in hippocampus facilitated contextual fear memory	NR	Walters et al., 2017
	FTO	KD	NA	KD of FTO in medial prefrontal cortex results in increased fear memory consolidation	*Rab33b, Arhgap39, Arhgef17, Crtc1, Gria1*, and *Crtc1*	Widagdo et al., 2016
	METTL3	cKO	*CaMKIIa-cre*	Decreased formation of hippocampus-dependent long-term memory	*Arc, Egr1, c-Fos, Npas4*, and *Nr4a1*	Zhang et al., 2018
	YTHDF1	KO	NA	Defects in learning and memory; impaired synaptic transmission and long-term potentiation	*Bsn* and *Camk2a*	Shi et al., 2018
	FTO	KO	NA	Impaired cocaine-induced behavioral activity and synaptic dopamine release	*Kcnj6, Grin1*, and *Drd3*	Hess et al., 2013
	METTL14	cKO	*Drd1-cre; Adora2a-cre*	Deficiency in striatum-mediated learning and dopamine signaling	*Tac1, Pdyn, Penk, Drd2, Homer1*, and *Cdk5r1*	Koranda et al., 2018

*KO, knockout; cKO, conditional knockout; KD, knockdown; NA, not applied; NR, not reported.*

### Differentiation and Neurogenesis

#### Cortex

During neuronal development, neurogenesis is a precisely orchestrated process (Kohwi and Doe, 2013). In the cerebral cortex, radial glia cells (RGCs), also known as neural stem cells (NSCs), are the principal progenitor cells that generate consecutive different types of neurons which further migrate to different layers. m^6^A modification has been shown to regulate this exquisitely timed process (Yoon et al., 2017; Wang Y. et al., 2018). When m^6^A modification was ablated in the nervous system using *Nestin-Cre;Mettl14^*f/f*^* conditional knockout (cKO) mice, the cell cycle of RGCs was prolonged and cortical neurogenesis extended into postnatal stages, causing brutal postnatal death (Yoon et al., 2017). These were due to the failure of m^6^A-dependent decay of mRNAs encoding proteins related to stem cell, cell cycle and neurogenesis in neural stem cells (Yoon et al., 2017). Another study also found cortical neurogenesis defects in *Nestin-Cre*;*Mettl14*^*f/f*^ cKO mice which were characterized with decreased cortical thickness and postnatal death (Wang Y. et al., 2018). In this study, m^6^A modification was found to mediate NSCs self-renewal, as deletion of *Mettl14* in NSCs led to reduced proliferation and premature differentiation, causing NSC pool loss and insufficient late-born neurons (Wang Y. et al., 2018). The underlying mechanism was that m^6^A modification could regulate histone modification, inhibiting proliferation-related genes and promoting differentiation-related genes (Wang Y. et al., 2018). These studies provided direct proof that m^6^A modification can regulate mouse embryonic cortical neurogenesis. However, the seemingly opposite mechanisms described in these two studies after using the same *Nestin-cre* to conditionally knock out *Mettl14* (“prolonged cell cycle and maintenance of radial glia cells” vs. “decreased proliferation and premature differentiation”) suggest that further exploration and clarification are needed. In addition, as *Nestin-cre* is generally expressed in the nervous system, how m^6^A modification specifically affects mouse cortical neurogenesis without affecting other areas in the brain needs further elucidation.

In addition, disrupting the recognition and reading of m^6^A modification can also phenocopy the effect on cortex neurogenesis (Li et al., 2018; Edens et al., 2019). Self-renewal and spatiotemporal neurogenesis of NSCs were severely affected in the cortex of *Ythdf2* knockout mice, causing retarded cortical development and lethality at late embryonic stages (Li et al., 2018). Both proliferation and differentiation abilities were decreased in *Ythdf2^–/–^* NSCs, which were indeed caused by the impaired clearance of m^6^A-modified genes (Li et al., 2018). Another reader protein involved in regulating cortical neurogenesis is fragile X mental retardation protein (FMRP) (Edens et al., 2019). The role of FMRP was to preferentially bind m^6^A-modified mRNAs and facilitate them to export from nuclear (Edens et al., 2019). Deletion of *Fmr1* cause nuclear retention of m^6^A-modified mRNAs associated with neural differentiation (Edens et al., 2019). Thus, *Fmr1* KO mice exhibited extended maintenance of NSCs into postnatal stages with delayed NSC cell cycle progression and differentiation.

#### Cerebellum

Unlike cortical neurogenesis that occurs in embryonic stages, the development of the cerebellum mainly begins postnatally. The cerebellum has generally higher m^6^A levels than the cerebral cortex (Ma et al., 2018). The expression of m^6^A modifiers (writers, erasers, and readers) is developmentally regulated and differentially expressed in different cell types and regions in the cerebellum (Ma et al., 2018). Similarly, the mRNAs in the cerebellum show dynamic methylation levels throughout the developmental stages (Chang et al., 2017; Ma et al., 2018). These findings demonstrate that m^6^A might be required for the development and function of the cerebellum.

Specific deletion of *Mettl3* in the mouse nervous system leads to cerebellar hypoplasia caused by increased apoptosis of newly generated cerebellar granule cells (CGCs) in the external granular layer (Wang C.X. et al., 2018). Due to the loss of m^6^A, the half-lives of mRNA associated with cerebellar development and apoptosis are extended. In addition, synapse-associated mRNAs show abnormal splicing after *Mettl3* depletion. Those events finally contribute to incorrect cell differentiation and cell death (Wang C.X. et al., 2018). Knockdown of METTL3 results in disorganized structures of Purkinje cells and glial cells in cerebellum (Ma et al., 2018). In addition, deletion of *Alkbh5* in mice exposed to hypobaric hypoxia leads to aberrant proliferation and differentiation due to the dysregulated mRNA nuclear export (Ma et al., 2018). Those findings together prove that m^6^A acts as a crucial regulator during cerebellar development.

### Axon Growth

Fat mass and obesity-associated was unexpectedly found expressed in the axons of mouse DRG neurons, which can dynamically regulate m^6^A modification on axonal mRNA (Yu et al., 2018). Despite the previous finding that demethylation occurs in nuclear speckles, m^6^A modification can be dynamically regulated in the highly compartmentalized subcellular structures such as axons. Axonally derived FTO regulates the level of m^6^A modification on *GAP-43* mRNA and further affects the local translation of *GAP-43* mRNA in axons, eventually controlling axon growth (Yu et al., 2018). This study is the first example of mRNA modification regulating local translation in axons.

### Axonal Guidance

Axon guidance cues provided by the floor plate enable the right pathfinding of commissural axons in the developing spinal cord (Colamarino and Tessier-Lavigne, 1995). Robo3.1 is one of the axon guidance receptors from Roundabout (Robo) family that facilitate midline crossing of commissural axons (Chen et al., 2008). The precise spatiotemporal expression of Robo3.1 has been found to be regulated by m^6^A modification (Zhuang et al., 2019). YTHDF1 can bind to *Robo3.1* mRNA in an m^6^A dependent manner and promote its translation (Zhuang et al., 2019). Specific deletion of YTHDF1 in commissural neurons using *Atoh1-cre;Ythdf1^*f/f*^ cKO* mice led to axon guidance defects (Zhuang et al., 2019).

### Axon Regeneration

Axon regeneration of mouse DRG neurons in the peripheral nervous system (PNS) requires *de novo* gene transcription and translation of regeneration-associated genes (RAGs) (Costigan et al., 2002; Mahar and Cavalli, 2018). Similar to other epigenetic mechanisms, such as DNA methylation (Weng et al., 2017) and histone modification (Gaub et al., 2011; Puttagunta et al., 2014), m^6^A modification has also been shown to participate in the activation of RAGs (Weng et al., 2018). Sciatic nerve lesion (SNL) substantially increases levels of m^6^A-modified transcripts *in vivo*. Those transcripts can be divided into three categories: transcripts encoding RAGs, injury-induced retrograde signaling molecules, and translation machinery components (Weng et al., 2018). Either loss of METTL14 or YTHDF1 can delay the injury-induced protein translation of RAGs, such as *Atf3*, and cause defective axon regeneration and function recovery (Weng et al., 2018). These findings indicate that m^6^A modification may affect response to pathological stimulus in the adult nervous system.

### Synapse

Low input m^6^A sequencing of mouse forebrain synaptosomes has revealed a synaptic m^6^A epitranscriptome (SME) in which 2921 synaptosomal transcripts are m^6^A-modified (Merkurjev et al., 2018). Transcripts in SME are most significantly enriched in central nervous system development. More than half of the genes in the SME overlapped with the synaptic transcriptome. Surprisingly, those genes are functionally annotated to synapse-associated functions, such as “synapse assembly,” “postsynaptic membrane,” “long-term synaptic potentiation.” In contrast, those hypomethylated transcripts in the synaptic transcriptome were mainly related to metabolic pathways. These findings suggest that m^6^A modification possibly regulates synapse formation and synaptic function. Dendrite localization of m^6^A writers, erasers, and readers was detected in mouse cortical pyramidal neurons in brain slices, which further indicates that m^6^A modification could be dynamically and locally regulated in synapses (Merkurjev et al., 2018). Either knockdown of YTHDF1 or YTHDF3 in cultured hippocampal neurons leads to abnormal dendrite spine morphology, reduced PSD95 clustering, decreased expression of GluA1, thus compromising synaptic transmission (Merkurjev et al., 2018).

### Adult Neurogenesis

Adult neurogenesis occurs (yet still in debate) limitedly, and has been shown to be related to neurological and psychiatric disorders (Apple et al., 2017; Kempermann et al., 2018). m^6^A has also been reported to function in adult neurogenesis. FTO is expressed in adult neural stem cells (aNSCs), and deletion of *Fto* reduces the proliferation and neuronal differentiation of aNSCs (Li et al., 2017; Cao et al., 2020). This is due to the altered expression of several key components that are modified by m^6^A in the brain-derived neurotrophic factor (BDNF) pathway (Li et al., 2017) and the Pdgfra/Socs5-Stat3 pathway (Cao et al., 2020). On the other hand, depletion of METTL3 also inhibits the proliferation of aNSCs (Chen J. et al., 2019). The mRNA of histone methyltransferase *Ezh2* is modified by m^6^A (Chen J. et al., 2019). Upon deletion of *Mettl3*, the protein level of Ezh2 deceased, further causing reduced H3K27me3 levels (Chen J. et al., 2019). These studies showed that m^6^A modification is involved in adult neurogenesis. However, how writers and erasers work together under normal conditions to regulate adult neurogenesis still needs more investigation.

### Gliogenesis

Glia cells account for more than 50% of cells in the human brain (Nave, 2010; Rowitch and Kriegstein, 2010). Oligodendrocytes and astrocytes are the two major macroglial cells derived from the neuroepithelium (Rowitch and Kriegstein, 2010). Oligodendrocytes are responsible for the myelination of axons. m^6^A modification has been shown to control the oligodendrocyte lineage progression. Specific deletion of *Mettl14* in developing oligodendrocyte lineage cells or in postmitotic oligodendrocytes leads to loss of mature oligodendrocytes and hypomyelination (Xu H. et al., 2020). This is because the loss of METTL14 results in abnormal splicing of many mRNAs which encode proteins associated with the myelinating process, such as protein tyrosine phosphate receptor type Z1 (Ptprz1) and neurofascin 155 (NF155) (Xu H. et al., 2020). Another study discovered a novel m^6^A reader, Proline-rich coiled-coil 2A (PRRC2A), which regulates oligodendrocyte specification and myelination (Wu et al., 2019). Deletion of *Prrc2a* in oligodendrocyte progenitor cells (OPCs) leads to hypomyelination, locomotive and cognitive defects in mice. Interestingly, PRRC2A directly stabilizes the *Olig2* mRNA in an m^6^A-dependent manner. Olig2 is known to regulate OPC specification, differentiation and myelination (Lu et al., 2002; Zhou and Anderson, 2002). However, the mechanism of how PRRC2A stabilizes m^6^A-modified mRNA remains unclear.

In addition to oligodendrocytes, m^6^A modification also functions in the gliogenesis of astrocytes, which provide structural support, modulate synaptic transmission, and maintain the blood-brain barrier (Rowitch and Kriegstein, 2010). Global deletion of *Mettl14* in the mouse nervous system significantly reduces astrocytes in the cortex (Yoon et al., 2017). The scaffold organization pattern provided by Bergmann glia (a highly diversified type of astrocytes) in the mouse cerebellum is abolished after deleting *Mettl3* in the nervous system (Wang C.X. et al., 2018). As for the m^6^A readers, *in vitro* differentiation assay of neurospheres showed that deletion of *Ythdf2* in neural stem/progenitor cell (NSPC) caused dramatic reduction of GFAP^+^ astrocytes (Li et al., 2018). These studies indicate that m^6^A modification also controls the production of astrocytes. However, the underlying mechanism needs further investigation.

### Learning and Memory

Learning and memory require coordinated regulation of gene expression and protein translation. The substantial increase of m^6^A level from the embryonic brain to the adult brain (Meyer et al., 2012) suggests that the dynamic m^6^A epitranscriptome could be involved in the regulation of the advanced brain functions.

Fat mass and obesity-associated is highly expressed in the dendrites and synapses of mouse CA1 hippocampal neurons (Walters et al., 2017). Interestingly, the expression of FTO protein decreased in the synaptic fraction, not the nuclear fraction of hippocampus 0.5 h after contextual fear conditioning, indicating that behavioral training-induced memory preferentially decreases FTO levels near synapses (Walters et al., 2017). As expected, with the decrease of FTO, the m^6^A level on mRNA is significantly increased. Knockdown of FTO in hippocampus facilitated contextual fear memory, suggesting that synaptic FTO could normally restrict memory formation and experience-induced increase of m^6^A modification could enhance memory formation (Walters et al., 2017). Another study also shows that cue fear conditioning increases m^6^A level in mouse medial prefrontal cortex (mPFC) (Widagdo et al., 2016). Knockdown of FTO in mPFC results in increased fear memory consolidation (Widagdo et al., 2016). These studies demonstrate that experience or behavior-induced upregulation of m^6^A modification might participate in the regulation of memory. However, as FTO was also reported to preferentially demethylate m^6^A_m_ (Mauer et al., 2017) and m^6^A_m_ participates in fear memory (Engel et al., 2018), the possibility that m^6^A_m_ may contribute to some of the phenotypes cannot be ruled out.

The study of m^6^A writer METTL3 provides direct evidence that m^6^A modification regulates memory formation. Using *CaMKIIa-cre;Mettl3^*f/f*^* cKO mice, specific deletion of METTL3 in the forebrain excitatory neurons decreases the formation of hippocampus-dependent long-term memory without changing short-term memory and learning ability when adequate training is provided (Zhang et al., 2018). The hippocampus-dependent memory consolidation ability exquisitely relies on the function of METTL3, as the expression of METTL3 in wild-type (WT) mice positively associates with the learning efficacy and overexpression of METTL3 facilitates long-term memory consolidation (Zhang et al., 2018). The formation of long-term memory requires *de novo* protein synthesis of immediate early genes (IEGs), such as *Arc*, *Egr1*, *c-Fos*, *Npas4*, and *Nr4a1*. By affecting the m^6^A levels on those IEGs, METTL3 eventually promotes their translation, thus enhancing memory (Zhang et al., 2018).

Regarding the roles of m^6^A reader protein, YTHDF1 was reported to be required in the process of m^6^A enhanced learning and memory in the hippocampus (Shi et al., 2018). *Ythdf1* mRNA is preferentially located in the mouse hippocampus (Lein et al., 2007), suggesting that it might be involved in learning and memory. When YTHDF1 is ablated entirely from the hippocampus, hippocampus histology, neurogenesis, motor ability, and emotional state are not altered in *Ythdf1* KO mice (Shi et al., 2018). However, by compromising basal synaptic transmission and long-term potentiation (LTP), the learning and memory abilities of *Ythdf1* KO mice in Morris water maze (MWM) and contextual fear conditioning tests are impaired (Shi et al., 2018). Restoring the expression of YTHDF1 in the hippocampus of *Ythdf1* KO mice can successfully rescue the synaptic and behavioral defects (Shi et al., 2018). Further analysis of the underlying molecular mechanism showed that YTHDF1 preferentially recognizes m^6^A modified mRNAs and facilitates their translation in a neuronal-stimulus-dependent manner. More interestingly, YTHDF1 could translocate into the postsynaptic density (PSD) fraction to facilitate protein synthesis locally in synapses of the hippocampus in response to fear conditioning, thus promote synaptic plasticity and memory formation (Shi et al., 2018).

Learning and memory-related synaptic plasticity requires local translation at synapses (Wang et al., 2009). Due to the dynamic SME (Merkurjev et al., 2018) and the localization of m^6^A writers, erasers, and readers in synapses, it’s highly likely that m^6^A-dependent local translation of synaptic mRNAs is the central event that occurs in synapses in response to stimuli.

Besides the forebrain, m^6^A modification also affects synaptic transmission in the midbrain and striatum. It has been shown that FTO can regulate the activity of the dopaminergic (DA) signaling in the mouse midbrain, which controls complex behaviors (Hess et al., 2013). Deletion of *Fto* attenuates neuronal activity controlled by dopamine D2-like receptor and behavioral responses (Hess et al., 2013). Compared with WT mice, *Fto-*deficient mice showed impaired cocaine-induced behavioral activity and synaptic dopamine release (Hess et al., 2013). Transcriptome-wide m^6^A sequencing showed that the m^6^A level of many genes involved in the DA signal pathway is increased in *Fto-*deficient mice. In the adult mouse striatum, specific deletion of *Mettl14* in two distinct but related types of neurons, striatonigral and striatopallidal neurons, leads to deficiency in striatum-mediated learning and dopamine signaling without affecting cell numbers and morphology (Koranda et al., 2018). Interestingly, neuronal and synaptic mRNAs are downregulated in either type of neurons after deletion of *Mettl14*, while upregulated mRNAs are mainly associated with translational regulation and metabolism (Koranda et al., 2018). These m^6^A-dependent gene regulation increases neuronal excitability and decreases spike frequency adaptation, which finally attenuates striatum-mediated learning and behavioral performance (Koranda et al., 2018). Considering activity-dependent synaptic protein synthesis is vital to synaptic plasticity and learning, it is important to decipher how m^6^A readers are involved in this process to spatial temporally regulate protein synthesis in response to neuronal activities.

## m^6^A in Neurological Disorders and Injuries

### Alzheimer’s Disease

Transcriptome-wide sequencing of human and mouse brains showed that m^6^A modification is spatiotemporally regulated during neurodevelopment and aging (Shafik et al., 2021). Increased m^6^A sites are observed as age increases. The dynamically regulated m^6^A sites are enriched in alternatively untranslated regions of genes involved in aging-related pathways (Shafik et al., 2021). Alzheimer’s disease (AD) is the most common form of dementia among elderly people (Bateman et al., 2012). The m^6^A levels of many transcripts involved in AD-associated pathways are decreased in the brain of a familial AD mouse model (5XFAD) (Shafik et al., 2021). In contrast, m^6^A levels are elevated in the cortex and hippocampus of APP/PS1 transgenic mice, another AD mouse model, compared with WT mice (Han et al., 2020). Interestingly, the expression of METTL3 increased, and FTO is decreased in the APP/PS1 mice (Han et al., 2020). These studies show that m^6^A modification is involved in AD. However, the mechanism by which m^6^A regulates the progression of AD remains almost unknown.

### Parkinson’s Disease

Parkinson’s disease (PD) is a common neurodegenerative disorder characterized by the early prominent death of dopaminergic neurons (Lees, 2017). The global m^6^A levels of mRNAs are decreased in the striatum of the PD rat brain and a cellular PD model (6-OHDA-induced PC12 cells), which is mainly due to the increase of FTO protein (Chen X. et al., 2019). The decrease of m^6^A level could induce the expression of *N*-methyl-*D*-aspartate (NMDA) receptor 1, and increase oxidative stress and Ca^2+^ influx, which finally leads to dopaminergic neuron apoptosis (Chen X. et al., 2019). Conversely, knockdown of FTO in PC12 cells decreases NMDAR1 expression and exhibits anti-apoptosis effect (Chen X. et al., 2019). This study suggests that m^6^A modification via FTO may play a crucial role in the pathogenesis of PD.

### Ischemia/Reperfusion Injury

Ischemic stroke is a severe neurological disease, which is a leading cause of disability in humans (Wang et al., 2017). Cerebral ischemia/reperfusion (I/R) injury rapidly triggers different types of programmed cell death in neurons. Several studies have shown that m^6^A modification was involved in I/R injury (Si et al., 2020). The expression of METTL3 is significantly decreased at the reperfusion injury period. Decreased m^6^A level leads to the reduction of miR-335 and stress granule formation (Si et al., 2020). Therefore, by upregulating the expression of miR-335, METTL3-mediated m^6^A modification can normally promote stress granule formation and improve cell survival of neurons. Contradictorily, another study reported increased m^6^A levels after I/R injury (Xu K. et al., 2020). The expression of m^6^A erasers, ALKBH5 and FTO, are decreased but not writers. Overexpression of m^6^A erasers can alleviate neuronal damage induced by I/R injury (Xu S. et al., 2020). A third study found that oxygen-glucose deprivation/re-oxygenation (OGD/R) increased METTL3-dependent m^6^A modification of long non-coding RNA D63785 (lnc-D63785), thus causing reduced expression of lnc-D63785 (Xu S. et al., 2020). Downregulation of lnc-D63785 further induces accumulation of miR-422a, which results in neuronal cell apoptosis (Xu S. et al., 2020).

Hypothermia is an effective therapeutic method to alleviate I/R injury (Callaway et al., 2015; Donnino et al., 2015). Hypoxia/reoxygenation (H/R) caused m^6^A-dependent increase of *PTEN* mRNA stability and consequently upregulation of its protein level, which could be reversed by hypothermia (Diao et al., 2020). Thus hypothermia could activate PI3K/Akt signaling to protect neurons from I/R-induced pyroptosis (Diao et al., 2020). Another study reported that the m^6^A reader YTHDC1 protects ischemic stroke through mediating *PTEN* mRNA degradation, which further promotes Akt phosphorylation and facilitates neuronal survival (Zhang Z. et al., 2020). These two studies demonstrate that m^6^A modification could modulate PTEN expression to regulate PI3K/Akt signaling in I/R injury.

Taken together, all these studies demonstrated that m^6^A modification is involved in the process of I/R injury, which could provide potential therapeutic targets for I/R injury.

### Traumatic Brain Injury

Traumatic brain injury (TBI), one of the most severe types of injury, is a major public health threat (Majdan et al., 2017). After TBI, the mRNA and protein levels of METTL3 were significantly decreased in the hippocampus of mice, but not other writers and erasers (Wang et al., 2019). Correspondingly, the m^6^A level of RNA was downregulated in the hippocampus after TBI. Genome-wide m^6^A profiling identified that altered peaks of m^6^A-modified transcripts after TBI were mainly related to the regulation of the metabolic process (Wang et al., 2019). Metabolism alteration induced by brain injury could lead to long-term cognitive and neurological disabilities (McKenna et al., 2015). Therefore, this study indicates that m^6^A modification-induced metabolic alteration might be the underlying mechanism of TBI. Thus rectifying altered m^6^A level might be a potential therapeutic strategy for TBI.

### Pathological Pains

*N*^6^-methyladenosine modification has been shown to participate in both inflammatory and neuropathic pain (Li et al., 2020a; Zhang C. et al., 2020). The m^6^A levels of spinal mRNAs are significantly increased in Complete Freund’s Adjuvant (CFA)-induced chronic inflammatory pain mouse model, which shows strong thermal and mechanical hyperalgesia (Zhang C. et al., 2020). The upregulated m^6^A level is due to the increase of METTL3 in CFA-injected mice, which can modulate the pain sensitization by regulating m^6^A-dependent pri-miRNA processing. Meanwhile, another study reported that FTO contributes to nerve injury-induced neuropathic pain in the primary sensory neurons in DRG (Li et al., 2020a). Nerve injury activates the transcription factor Runx1, which can bind to *Fto* gene promoter and activate its expression but not m^6^A readers. Upregulated FTO then demethylates m^6^A modification on the *Ehmt2* mRNA encoding euchromatic histone lysine methyltransferase 2 and elevates its protein level, thus resulting in neuropathic pain symptoms. Conversely, knockdown of FTO could alleviate nerve injury-associated pain hypersensitivities (Li et al., 2020a). These two studies indicate that m^6^A modification regulates different pain responses through different mechanisms.

### Brain Tumor

*N*^6^-methyladenosine modification has been implicated in various types of cancer including brain tumor (Deng et al., 2018). Glioblastoma is the most common and severe brain tumor. The proliferation and tumorigenesis of glioblastoma stem cells (GSCs) require high expression of the m^6^A eraser ALKBH5 (Zhang et al., 2017). ALKBH5 demethylates *FOXM1* nascent transcript, maintaining expression of FOXM1 that preserves GSC properties (Zhang et al., 2017). Knockdown of ALKBH5 reduces proliferation of patient-derived GSCs (Zhang et al., 2017). In addition, knockdown of the m^6^A writers METTL3 and METTL14 significantly increases GSC-initiated tumor progression *in vivo* (Cui et al., 2017). Interestingly, treatment with MA2, an FTO inhibitor, inhibits GSC-initiated tumorigenesis and extends the lifespan of GSC-engrafted mice (Cui et al., 2017). Controversially, another two studies found that clinical aggressiveness of glioblastoma is related to increased expression of METTL3 (Visvanathan et al., 2018; Li et al., 2019). METTL3 promotes GSC stemness by enhancing SOX2 stability in glioblastoma, and METTL3 silencing inhibits tumor growth (Visvanathan et al., 2018). Knockdown of METTL3 suppresses aggressive and tumorigenic properties of GSCs by causing YTHDC1-dependent nonsense-mediated mRNA decay of *SRSF*, and subsequent decrease of SRSF protein level (Li et al., 2019). m^6^A modification also regulates neuroblastoma, another common malignant brain tumor (Cheng et al., 2020). MYCN is a genetic biomarker of high risk and poor outcome in neuroblastoma. m^6^A modification in the 3′UTR of *MYCN* promotes its interaction with miR-98, decreasing MYCN expression and inhibiting neuroblastoma progression (Cheng et al., 2020). These studies indicate that m^6^A modification could be a promising target for anti-brain tumor therapy.

## Conclusion and Perspectives

The nervous system is the most complex and diverse system, with exceptional capabilities that control higher cognitive and emotional functions. The development of the nervous system is a highly coordinated process in which epigenetic mechanisms exert significant effects by spatiotemporally regulating gene expression. Apart from DNA methylation and histone modifications, dynamic mRNA m^6^A modification provides an additional regulatory layer to regulate gene expression. As described above, m^6^A modification regulates the development and functions of the nervous system.

The higher function of the nervous system relies on synaptic plasticity. In response to stimuli, the nervous system undergoes extremely swift reactions to adapt its proteome. Neurons are highly compartmentalized cells, and local translation plays a central role in rapidly changing subcellular proteomes in response to extrinsic cues and stimuli. Accumulating evidence has suggested that m^6^A modification modulates the local translation of mRNAs in axons and synapses. This m^6^A-dependent local translation could be the principal mechanism that regulates the plasticity of the nervous system. This highlights the requirement of comprehensive studies of m^6^A modification and local translation of the nervous system. How m^6^A writers, erasers, and readers function together to spatiotemporally regulate local proteome needs more investigation.

Up to now, there are controversial findings regarding the functions of m^6^A reader proteins. As YTH proteins share very high similarity in the YTH domains, the mechanism of how these reader proteins select their target mRNA remains unknown. Therefore, it is crucial to deeply decipher the roles and mechanisms of reader proteins on how they divide jobs and coordinate to mediate m^6^A signaling.

Dysregulation of m^6^A modification causes neurological diseases. The involvement of m^6^A in neurological diseases and injuries provides new potential therapeutic targets for treatment. However, the roles of m^6^A in injury-induced neuronal diseases, psychiatric disorders, and aging-related neurodegenerative disorders are still far beyond understanding. In-depth studies of how m^6^A signaling modulates neuronal physiology and pathology in the adult brain are in great demand.

## Author Contributions

JY and S-JJ drafted and revised the manuscript. S-JJ conceived and designed the review. YS helped to edit and revise the manuscript. All authors read and approved the final manuscript.

## Conflict of Interest

The authors declare that the research was conducted in the absence of any commercial or financial relationships that could be construed as a potential conflict of interest.
